# Systems‐Level Optimization of Hybrid Produced Water Treatment Systems for Sustainable Oil and Gas Production: A Review of Current Technologies

**DOI:** 10.1002/gch2.202500575

**Published:** 2026-02-09

**Authors:** Omogbolade L. Adepitan, Oluwaseyi O. Alabi, Charles Deigh, Oluwatoyin Joseph Gbadeyan

**Affiliations:** ^1^ Department of Mechanical Engineering Lead City University Ibadan Nigeria; ^2^ Oando Energy Resource Nigeria Limited Port Harcourt Nigeria; ^3^ Department of Chemistry Durban University of Technology Durban South Africa

**Keywords:** environmental sustainability, membrane technologies, process optimization, produced water treatment, thermal and hybrid processes

## Abstract

Produced water (PW) is the largest and most complex waste stream generated during oil and gas production, posing significant environmental, operational, and regulatory challenges due to its high salinity, dispersed hydrocarbons, toxic organics, heavy metals, and naturally occurring radioactive materials. Although numerous studies have reviewed individual treatment technologies, a critical, systems‐level synthesis linking treatment performance, optimization strategies, and sustainability objectives across the produced water management chain remains lacking. This synthesis should encompass current technologies used in the treatment of produced water. This study presents a critical literature review of produced water treatment and management technologies reported in peer‐reviewed journals and selected industrial case studies over the past decade. The review synthesizes produced‐water treatment technologies across primary, secondary, and tertiary stages. It critically evaluates performance, energy and cost trade‐offs, and key operational constraints. The analysis identifies suitable pathways for reuse, reinjection, and zero‐liquid‐discharge applications. The review shows that no single technology can effectively address the wide variability in produced water composition. Instead, hybrid treatment trains integrating mechanical separation, membrane filtration, and thermal or oxidative polishing consistently outperform standalone systems in terms of robustness and water recovery. Practical optimization is governed primarily by pretreatment design, energy integration, and adaptability to fluctuating feed chemistry, rather than by isolated unit efficiency. Persistent gaps are identified, including limited full‐scale validation of emerging technologies (e.g., FO‐MD hybrids), insufficient life‐cycle and carbon‐footprint assessments, and underutilization of digital optimization and predictive control tools. The review finds that produced water treatment performance is maximized when technologies are designed and evaluated as integrated systems rather than isolated unit operations. Energy demand, fouling and scaling control, and pretreatment requirements emerge as the dominant constraints governing operational reliability and cost‐effectiveness. Hybrid treatment trains consistently outperform standalone processes in enabling reuse, reinjection, and zero‐liquid‐discharge applications. Significant gaps remain in the consistent application of sustainability metrics, life‐cycle assessment, and digital optimization tools across treatment systems.

## 1 Introduction

**FIGURE 1:**
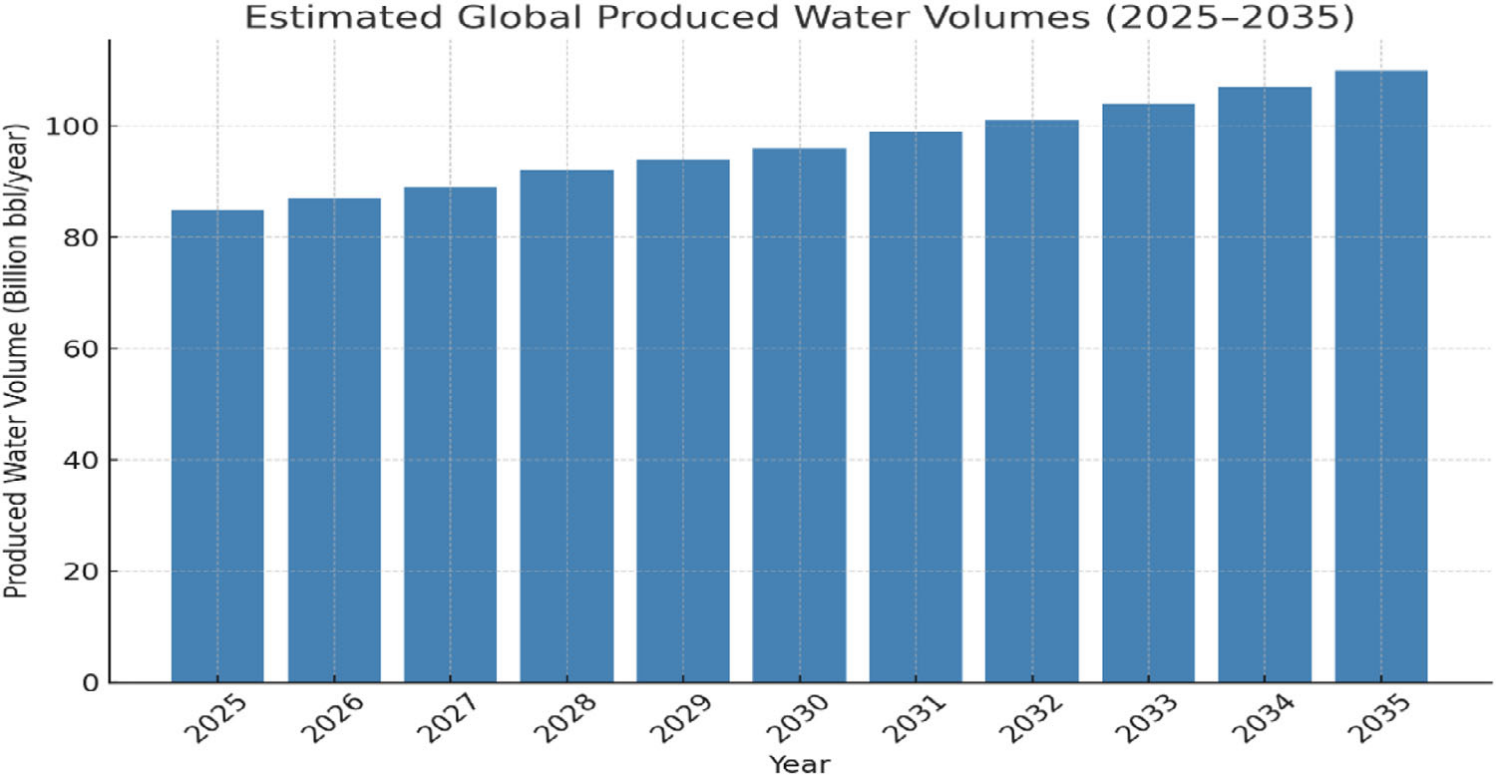
Estimation of PW by 2035 [1].

During crude oil exploration, an aqueous fluid often comes out to the surface along with oil and gas. The associated aqueous is Produced Water, often abbreviated as PW. The aqueous fluid known as Produced Water PW, has been tagged as the largest single waste stream in the oil and gas sector, which includes the formation of liquid (water) from many processes, like the flow from the injection return, fluid introduced by drilling operations. Its management spans on‐site separation, interim storage, treatment, reuse, deep well injection, and sometimes off‐site transfer [1, 2]. Globally, the amount (volume) of PW is enormous due to the increase in the demand for hydrocarbon‐based products as well as prolonged field life [3]. According to estimation, annually, about tens of billions of barrels are produced globally, with the United States alone producing approximately 25 billion bbl in 2021, and the total global reports are commonly given in are commonly reported in the multi‐10^9^–10^10^ barrels/yr range with regionally variable growth driven by unconventional development, water‐cut increases as fields mature, and expanding offshore activity [4]. There is interest in the reuse of PW due to the scarcity of fresh water, since indication shows that the trajectory from 2010 shows an increase in the production of basins. Figure 1 shows an estimation of the amount of produced water that would have been produced by the year 2035 [1].

Recent research and review studies have examined produced water management, primarily focusing on individual treatment technologies such as physical separation, membrane filtration, thermal desalination, biological treatment, and advanced oxidation processes. Recent articles have provided detailed assessments of membrane‐based desalination, particularly reverse osmosis and emerging membrane distillation systems, while others have evaluated thermal approaches for high‐salinity brines or biological processes for organic removal. More recent reviews have also discussed reuse opportunities and regulatory considerations for produced water discharge. Collectively, these studies have significantly advanced the understanding of individual unit processes and their performance limits.

Despite these contributions, existing reviews largely remain fragmented and technology‐centric, with limited integration of system‐level design logic, optimization strategies, and sustainability considerations across the full produced water management chain. Few studies critically compare hybrid treatment trains under varying feed compositions, operational constraints, and reuse or zero‐liquid‐discharge objectives. Moreover, optimization is often discussed qualitatively, without explicit linkage to pretreatment design, energy integration, life‐cycle impacts, or adaptability to fluctuating water chemistry. The role of digital optimization, predictive control, and data‐driven decision‐making in produced water systems also remains underexplored in the review literature.

**FIGURE 2:**
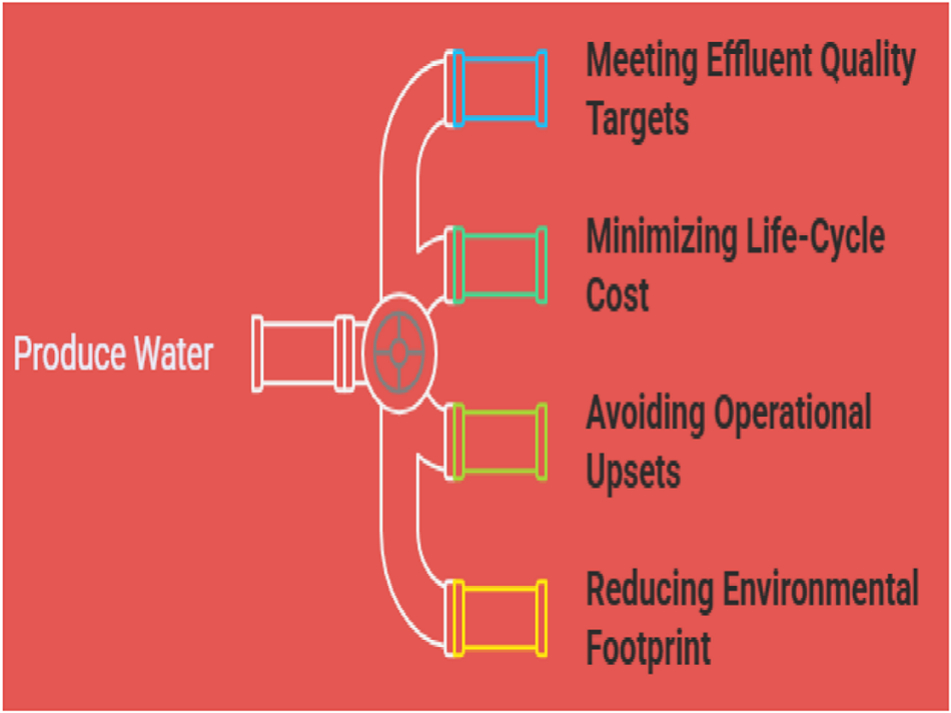
Optimizing for proper PW management.

The composition of PW is complex and solely depends on the sources or field and the life span of the well source [5]. Basically, the composition of PW varies, some contain a high concentration of salinity, that is total dissolved solids from a few thousand to >200 000 mg/L, dispersed and dissolved hydrocarbons (oil drops, BTEX, PAHs), dissolved and particulate organics (surfactants, biopolymers), elevated concentrations of major ions (Na^+^, Cl^−^, Ca^2+^, and Mg^2+^), heavy metals (e.g., Pb, Ni, Fe, and Ba), and naturally occurring radioactive materials (NORM, principally radium isotopes) [5, 6, 7]. The complexity of these compositions, which include the variable pH, temperature, and suspended solids, dictates the kind of technology that would be needed by the downstream for their pretreatment [8]. These focus on different critical areas, which are (a) meeting effluent quality targets appropriate to disposal or reuse, (b) minimizing life‐cycle cost (CAPEX + OPEX), (c) avoiding operational upsets (fouling, scaling, corrosion, NORM handling), and (d) reducing environmental footprint (energy, discharge impacts, waste brine) as seen Figure 2 with a pictorial illustration. Digitally driven optimization process modelling, dynamic control, and predictive maintenance increasingly appear as a high‐value enabler in modern PW systems [9, 10, 11].

However, there is a strong need to balance costs alongside energy consumption against their reuse. Recently, review studies emphasized two types of membranes, which are the membrane‐AOP hybrids and staged membranes with automated pretreatment yields; these are the best compromise between water quality, process stability, and overall cost effectiveness for many reuse cases [11, 12, 13, 14, 15]. Optimization of the process of design of PW treatment does not just purify PW but also a potential source of water for reinjection, enhanced oil recovery, agricultural or industrial reuse (in water‐stressed regions), or as a feedstock for mineral recovery (salts, lithium/rare earths in some basins) [13]. Economics and regulatory acceptance determine which reuse paths are viable. In contrast, desalination alongside reuse can reduce freshwater withdrawals; the cost of achieving potable or irrigation standards for very high‐TDS brines remains prohibitive in many contexts. Techno‐economic studies and pilot demonstrations are therefore crucial to show when resource recovery pays [15].

Another concern in the design and optimization of produced water is the operational constraints coupled with the environmental risks. The formation of scale (fouling) containing several cations, such as Ca/Mg/Barite/Strontium salts, the deposition of also organic matter, emulsion stability, NORM handling, and variable feed chemistry cause unplanned downtime and higher costs, which are part of the operational constraints. Environmental concerns include the impacts of the disposed brine, contents of the discharged water like organics and metals, and the potential mobilization of radioisotopes [15, 16, 17]. To optimize the design and management of these, a holistic monitoring which must include anti‐scalant strategies, adaptive chemical dosing, and proper disposal and handling of effluent in the environment, must be the top priority. Sophisticated tools for design and optimization can be employed to address all these issues [16]. Life‐cycle assessment (LCA) and integrated techno‐economic‐environmental optimization are increasingly recommended for choosing between partial treatment for EOR, full desalination for reuse, or ZLD for minimal discharge [17]. In response to these gaps, this review aims to: (i) critically synthesize current produced water treatment technologies from a systems‐level perspective rather than as isolated unit operations; (ii) evaluate optimization drivers and constraints, including pretreatment requirements, energy integration, fouling and scaling control, and operational flexibility; (iii) assess the suitability of standalone and hybrid treatment trains for disposal, reuse, and zero‐liquid‐discharge applications; and (iv) identify key research and implementation gaps related to sustainability metrics, life‐cycle impacts, and digital optimization. The scope of the review encompasses primary, secondary, and tertiary treatment technologies, with particular emphasis on membrane‐based, thermal, hybrid, biological, and advanced oxidation systems relevant to modern oil and gas operations.

This review employed a structured literature review protocol using peer‐reviewed articles retrieved from Scopus, Web of Science, and ScienceDirect. Publications published between 2014 and 2024 were considered to capture recent advances in produced water treatment technologies for oil and gas operations. Targeted keyword searches focused on hybrid treatment systems, systems‐level optimization, and sustainability assessment. Inclusion criteria prioritized studies addressing integrated or hybrid produced water treatment systems with quantitative performance or optimization analysis. Irrelevant, non‐peer‐reviewed, and single‐process studies were excluded. Selected articles were screened through title, abstract, and full‐text evaluation, followed by qualitative synthesis to identify dominant technological trends and optimization strategies.

## 2 Characteristics of Produced Water

One of the largest waste streams generated from crude oil exploration and production is produced water (PW). In a miniature field of oil exploration and production, it is estimated that about 5 barrels of produced water are generated globally, with a ratio as high as 10:1 of water‐to‐oil. This high‐volume ratio makes produced water management optimization a serious concern in the crude oil production sector [18]. The composition of produced water is a heterogeneous, highly complex, and variable mixture. This complexity in produced water is influenced by reservoir geology, depth of well, hydrocarbon classification, well's age, and production practices. Unlike conventional industrial effluents, produced water can contain both dissolved and suspended solids, dispersed hydrocarbons, dissolved gases, organic acids, heavy metals, and even naturally occurring radioactive materials (NORMs) [18]. Such variability means that treatment solutions effective in one field may be inadequate in another, necessitating tailored design and optimization strategies [9].

However, the heavy metals, harmful hydrocarbons, radionuclides, and salinity found in this produced water are all environmental threats to the aquatic organisms and the ecosystem at large [18]. Regulatory agencies worldwide, including the U.S. Environmental Protection Agency (EPA) and other environmental agencies in Europe, have come up with regulations guarding the discharge of some effluents in oil exploration and production, such as radionuclides, benzene, oil and grease, which undermine the effective management and fundamental composition of produced water. The characterization of produced water helps properly design and optimize a treatment system that can predict environmental impacts and evaluate reuse or reinjection potential. Among these compositions of produced water, the most significant characteristics are salinity, total dissolved solids (TDS), oil and grease, organic compounds (BTEX, phenols, PAHs), heavy metals, and radionuclides. Each of these parameters has distinct implications for treatment complexity, regulatory compliance, and environmental safety [9].

### 2.1 Salinity

**FIGURE 3:**
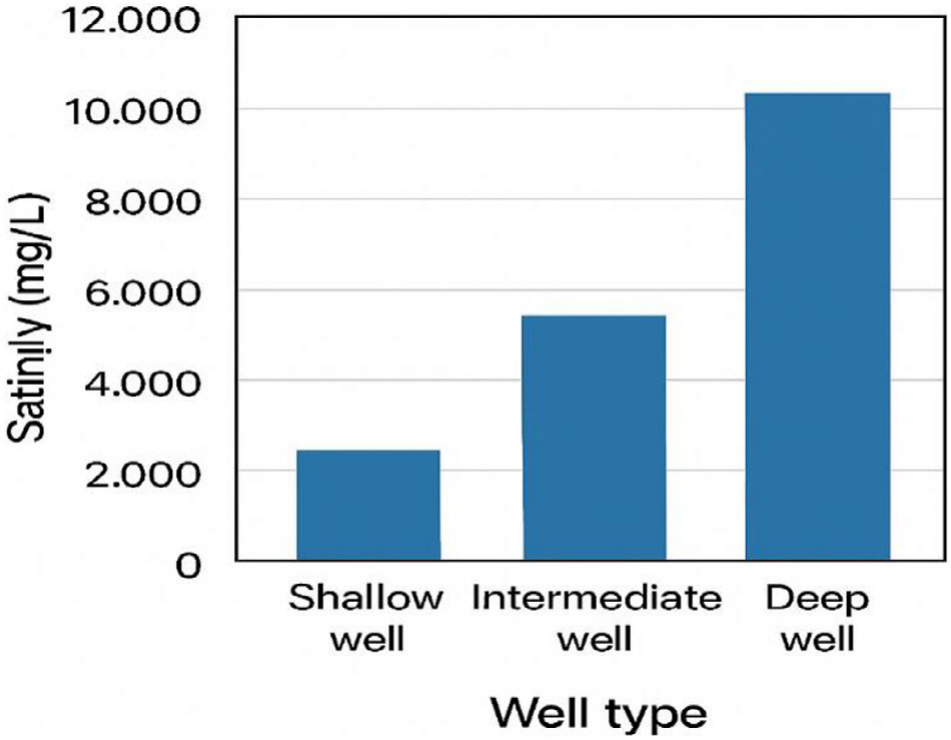
Salinity of produced water based on well type [8].

One of the most critical properties of produced water that influences the design and selection of an appropriate technological method, as well as disposal methods, is salinity. The salinity level of produced water can range between <1,000 mg/L as NaCl to hyper‐saline levels over 200 000 mg/L, which is significantly higher than seawater with approximately 35 000 mg/L. Salinity is mainly attributed to the dissolution of salts, primarily sodium chloride, calcium and magnesium salts, and bicarbonates, from the surrounding rock formations [2, 8, 19, 20]. However, produced water with a higher level of salinity tends to have unpleasant physical behavior like density and scaling, and also reduces the biological treatment processes. It can also lead to corrosion in pipelines and equipment, thereby influencing operational costs [8]. The salinity of produced water also depends on the type of well; the shallow well (<50–100 ft) produces the lowest salinity level, while the deep well (>100 ft) has the highest level of salinity. Figure 3 illustrates the amount of salinity based on the types of wells [20].

### 2.2 Total Dissolved Solids (TDS)

Total Dissolved Solids (TDS) is closely related to salinity and serves as a quantitative indicator of the dissolved ionic load in produced water. The concentration of TDS varies significantly across basins, reflecting geological diversity. For instance, produced water from coal‐bed methane reservoirs often contains TDS as low as 500–2000 mg/L, while that from conventional oil fields may range between 10 000–100 000 mg/L, and deep gas reservoirs may exceed 200 000 mg/L [21]. High TDS levels influence treatment options, as many conventional desalination technologies (e.g., reverse osmosis) are challenged at concentrations above 40 000–50 000 mg/L. Table 1 illustrates typical TDS ranges by reservoir type.

**TABLE 1:** Typical TDS concentrations in produced water by reservoir type [21].

Reservoir type	TDS range (mg/L)	Remarks
Coalbed Methane	500 – 2000	Often low salinity; may be reusable locally
Onshore Oil Fields	10 000 – 100 000	Highly variable; influenced by geology
Offshore Oil & Gas	20 000 – 150 000	Seawater intrusion can contribute
Deep Gas Reservoirs	100 000 – 200 000+	Hypersaline treatment is technically costly

### 2.3 Oil and Grease

The dispersed and dissolved heavy hydrocarbons in the aqueous phase present in produced water are referred to as oil and grease. The concentrations of oil and grease are between 2 and 565 mg/L, which depend on reservoir characteristics and efficient separation [22, 23]. These hydrocarbons are generally classified into dispersed oil droplets, dissolved hydrocarbons, and surface‐active agents. A higher level of oil and grease content is problematic to water treatment as it causes fouling, reduces dissolved oxygen by elevating Chemical Oxygen Demand (COD) [24, 25, 26]. Many regulatory agencies, such as the U.S. Environmental Protection Agency (EPA), set discharge limits for oil and grease at 29 mg/L (monthly average) for offshore operations [25].

### 2.4 Organics (BTEX, Phenols, and Others)

The composition of produced water includes many which, including a wide variety of organic compounds like Benzene, Toluene, Ethylbenzene, and Xylene (BTEX) and phenols, which are the most dominant, due to their solubility, toxicity, and persistence. Table 2 shows the amount in mg/L of concentrations of some of the significant organic compounds. For instance, the amount of BTEX in a typical produced water is between 0.1 to 200 mg/L, with a larger percentage of benzene, with environmental concerns due to its carcinogenic nature. Also, compounds of phenols are present in varying concentrations between 0.1 to 30 mg/L, which affect the aquatic animal due to their toxic nature [27, 28, 29, 30]. In addition, polycyclic aromatic hydrocarbons (PAHs), which are higher molecular weight hydrocarbons, are also present in traceable amounts in produce <1 mg/L. These compounds pose challenges to treatment due to their recalcitrant nature and require advanced oxidation or adsorption processes for removal [13, 31].

**TABLE 2:** Typical organic compounds in produced water [13, 30, 31].

Compound class	Typical concentration range (mg/L)	Environmental concern
Benzene	0.01 – 200	Carcinogenic; soluble and mobile
Toluene	0.01 – 50	Neurological effects: moderately soluble
Ethylbenzene	0.01 – 20	Toxic to aquatic life
Xylene (o, m, p)	0.01 – 25	Affects CNS; persistent
Phenols	0.1 – 30	Acute aquatic toxicity; odor issues
PAHs	<0.1 – 1.0	Persistent, bioaccumulative, mutagenic

### 2.5 Heavy Metals

During the production of crude oil, heavy metals are leached from the walls of the reservoir, thereby increasing the concentration of heavy metals in the produced water, heavy metal such as barium, cadmium, chromium, lead, arsenic, iron, and zinc [32]. Barium sulfate, which causes scale formation, is found in larger amounts due to barium ranging between 1 mg/L to 300 mg/L, as shown in Table 3. Other toxic metals like cadmium, lead, and mercury are found in trace levels (µg/L–mg/L), pose significant ecological and health risks due to bioaccumulation [33]. These metals contribute to long‐term environmental contamination if not adequately removed before discharge.

**TABLE 3:** Heavy metals in produced water [32, 33].

Metal	Typical range (mg/L)	Environmental/Operational concern
Barium	1 – 300	Scale formation; aquatic toxicity
Iron	0.1 – 100	Scaling, corrosion, turbidity
Zinc	0.1 – 50	Toxic to aquatic organisms
Lead	0.01 – 10	Carcinogenic, bioaccumulative
Cadmium	0.001 – 1	Highly toxic, accumulates in biota
Arsenic	0.01 – 5	Human health risk (carcinogenic)
Mercury	<0.001 – 0.1	Extremely toxic, persistent, bioaccumulative

### 2.6 Radionuclides (Naturally Occurring Radioactive Materials (NORMs))

Natural Occurring Radioactive Materials (NORMs) are often present in produced water from subsurface formations. Radioactive isotopes like Ra‐226, Ra‐228, uranium, and thorium. Radium isotopes are the most common radionuclides, including isotopes of radium, which are soluble in produced water and are of particular concern due to their carcinogenic potential and ability to accumulate in scales and sludges. Concentrations vary by geology but are typically found in the range of 1–10 000 pCi/L (picocuries per liter), which is well above the U.S. EPA drinking water limit of 5 pCi/L for combined Ra‐226 and Ra‐228. Radionuclides pose not only environmental hazards but also occupational risks to workers during the handling and disposal of produced water and associated sludge [34].

Produced water (PW) is characterized by its highly variable such as extreme salinity and TDS, substantial oil and grease content, and the presence of hazardous organic pollutants (BTEX, phenols, PAHs), heavy metals, and radionuclides. These parameters directly influence the selection of treatment and disposal technologies, regulatory compliance, and the economic feasibility of management options. The chemical complexity makes produced water one of the most challenging industrial effluents to manage sustainably, underscoring the need for integrated treatment strategies and advanced monitoring systems.

## 3 Current Treatment Technologies

There are modern technologies of PW; these families of modern technologies fit into different PW characterizations. Some of these properties are the mechanical or physical separation (e.g. gravity separators, hydrocyclones, API separators, induced gas flotation) for bulk oil removal, solids removal and conditioning properties (e.g. centrifuges, filtration, coagulation/flocculation) which serves as pretreatment; membrane systems (e.g. micro/ultra/microfiltration, reverse osmosis, membrane distillation, electrodialysis) for desalination and oil or solute removal; adsorption and ion‐exchange for organics and specific ions; advanced oxidation processes (AOPs), electrochemical oxidation, and biological systems for refractory organics; and finally, thermal methods and evaporators for high‐salinity streams and zero‐liquid discharge (ZLD) cases. Hybrid trains that combine these elements are the norm for challenging feeds. Performance tradeoffs include energy intensity (thermal and RO work), fouling and scaling propensity, secondary wastes (spent media, brine), and footprint [11, 12, 13, 14, 15]. Membrane systems and advanced oxidation, especially when robust pretreatment is involved in stage trains, due to high removal efficiencies and compactness, but there are some limiting factors to be scaled, such as concentrate management, fouling, and chemical compatibility [12]. Advanced Oxidation Processes (AOPs) and electrochemical methods are effective in the desalination units and can also mineralize organics [13]. Treating produced water technologically can be classified into three stages, namely, primary, secondary, and tertiary processes, which are based on the amount and kind of contaminants and the quality of effluent desired. Removing particles like large particles, suspended solids, and dispersed oil can be done using physical techniques as the primary method. While the introduction of hydrocyclones and gravity separation is used in the secondary treatment for effective and more cost‐effective results in the onshore and offshore operations to achieve oil‐in‐water concentrations below regulatory limits [35, 36, 37]. However, the focus of the secondary categories is the removal of more contaminants, like dissolved organics, finer emulsified oil, and some inorganic constituents, using coagulation‐flocculation, filtration, and other biological processes. In particular, membrane bioreactors (MBRs) and constructed wetlands have shown increasing promise for biodegradable organic removal and partial desalination, especially for reuse or discharge compliance [38]. At the tertiary level, advanced physicochemical and hybrid systems are employed to remove dissolved salts, toxic metals, and refractory organic compounds. Technologies such as reverse osmosis (RO), nanofiltration (NF), ion exchange, and advanced oxidation processes (AOPs) are increasingly adopted for high‐quality water recovery. Emerging methods like membrane distillation, electrocoagulation, capacitive deionization, and forward osmosis are being explored for improved energy efficiency and lower fouling potential. However, high salinity, complex organic mixtures, and scaling tendencies continue to limit the efficiency and lifespan of these systems [39, 40]. Current research, therefore, focuses on developing integrated and modular treatment trains, combining physical, chemical, and biological approaches to achieve sustainable, cost‐effective, and field‐adaptable produced water management.

### 3.1 Primary Treatment (Bulk Oil and Solids Removal)

**FIGURE 4:**
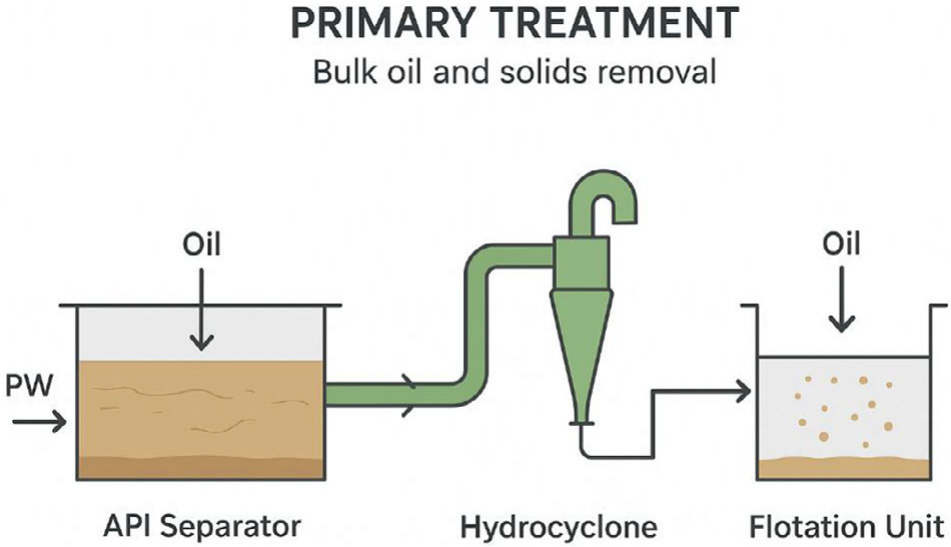
Primary treatment (Bulk oil and solid removal).

Primary treatment is the first and most fundamental stage in produced water management systems. Its main objective is the removal of free oil, grease, suspended solids, and coarse particulates that are physically separable from the water phase, as shown in Figure 4. This step significantly reduces the organic load and turbidity of the produced water before it undergoes secondary or tertiary treatment [41]. The overall performance of this stage determines how efficient the whole process is, as excessive oil and solids can foul membranes, deactivate catalysts, or inhibit biological activity downstream. Primary treatment methods rely largely on gravity separation and mechanical forces, exploiting differences in density, particle size, and hydrodynamic behavior between oil, water, and solids. The major technologies employed include API separators, hydro‐cyclones, and flotation units [42].

#### 3.1.1 API Separators

**FIGURE 5:**
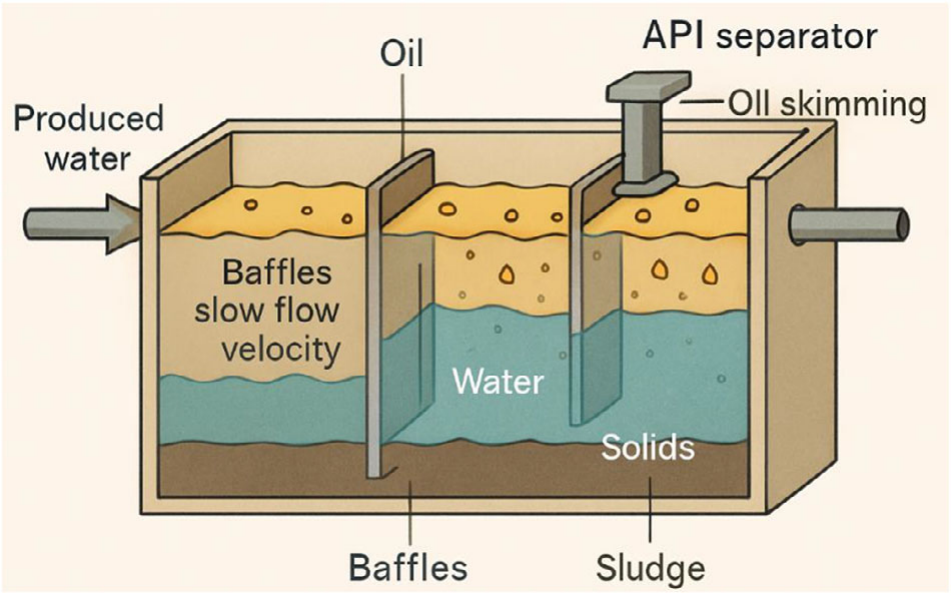
API unit.

One of the oldest technologies used in produced water treatment is the American Petroleum Institute (API) separator. This API separator is based on Stokes’ law of sedimentation and a gravity‐based system designed to remove free oil droplets >150 µm and settleable solids >100 µm. Table 4 shows a typical design and performance characteristics of API Separators [43, 44, 45]. The separator consists of a long rectangular tank with baffles that slow the flow velocity, allowing oil droplets (less dense) to rise to the surface and solids (denser) to settle at the bottom. After this process, the oil in the separator is skimmed off the top and recovered for potential reuse, after which, the settled solids are periodically removed as sludge. Figure 5 shows API unit

**TABLE 4:** Typical design and performance characteristics of API separators [43, 44, 45].

Parameter	Typical value/range	Remarks
Oil Droplet Size Removed	>150 µm	Effective for free oil only
Hydraulic Retention Time (HRT)	30–60 min	Dependent on flow and tank geometry
Oil Removal Efficiency	40%–70%	Improves when combined with coalescers
Solids Removal Efficiency	30%–50%	Limited for fine or colloidal particles
Typical Application	Onshore oil fields, refineries	Usually, before hydrocyclones or flotation units

American Petroleum Institute (API) separators are often produced from steel or concrete and are easy to construct, operate, and maintain. Their design efficiency depends on the residence time, flow rate, temperature, and viscosity of the produced water. Despite their simplicity, they can achieve oil removal efficiencies of 40%–70%, which makes them suitable as the first stage of treatment before finer separation methods [7, 46, 47, 48, 49].

#### 3.1.2 Hydrocyclones

**FIGURE 6:**
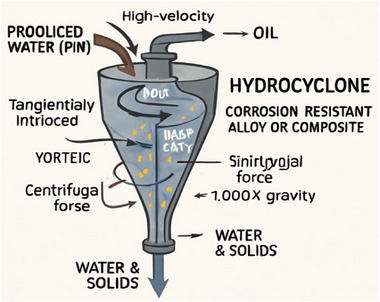
Hydrocyclones for produced water treatment.

Another primary treatment of produced water is the hydrocyclones. The hydrocyclones are compact and are based on the principle of force (centrifugal force) with high‐throughput devices that separate oil droplets from water, unlike the API, which is based on gravity. Produced water (PW) is tangentially introduced into a conical chamber at high velocity, generating a vortex. The centrifugal force, which is about 1,000 times gravity, drives denser water and solids to the wall of the cyclone and downward, while lighter oil droplets migrate to the center and exit through an overflow outlet. Figure 6 shows hydrocyclones for Produced Water treatment [44, 45, 46, 47].

Oil droplets between the ranges of 10 – 50 µm are efficiently removed by hydrocyclones and can handle large volumes in a small footprint, making them particularly suitable for offshore platforms, where space is limited [45]. Table 5 shows the characterization and design of a typical hydrocyclone. The materials used in the fabrication of a typical hydrocyclone must have a good corrosion resistance capability, like steel, aluminum or composite materials and require minimal moving parts, thus offering mechanical simplicity [46]. However, the efficiency of a typical hydrocyclone depends solely on the size of oil droplets, viscosity and a steady flow rate, as their efficiency drops with high viscosity and unsteady flow rate [44]. Hydrocyclones are typically installed downstream of API separators to further polish the effluent before secondary treatment.

**TABLE 5:** Typical design and performance characteristics of hydrocyclones [45, 46].

Parameter	Typical value/range	Remarks
Oil Droplet Size Removed	10–50 µm	Moderate separation efficiency
Operating Pressure	1–6 bar	Higher pressure increases centrifugal force
Oil Removal Efficiency	60%–90%	Depends on inlet pressure and flow stability
Space Requirement	Compact (<1 m^2^ per unit)	Ideal for offshore installations
Typical Application	Offshore oil & gas, compact systems	Often integrated with flotation units

#### 3.1.3 Flotation Units

**FIGURE 7:**
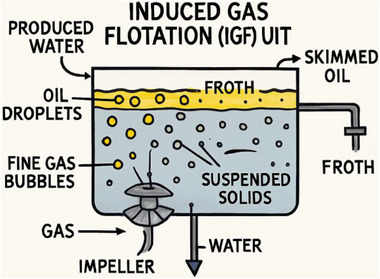
Floating unit for PW treatment.

After the application of API separation or hydrocyclone treatment, the treatment process is the Flotation systems, which is widely applied to remove residual dispersed oil and suspended solids [44]. These systems use fine gas bubbles (air, nitrogen, or natural gas) to attach to oil droplets and suspended solids, increasing their buoyancy and allowing them to rise rapidly to the surface for skimming, as shown in Figure 7, with Table 6 showing the characterization of the design [50]. The most common designs are Induced Gas Flotation (IGF) and Dissolved Air Flotation (DAF) units.

**TABLE 6:** Typical design and performance characteristics of flotation units [50].

Parameter	Typical value/range	Remarks
Oil Droplet Size Removed	5–10 µm	Effective for fine and emulsified oil
Gas Type	Air, Natural Gas, or Nitrogen	Choice depends on safety and process conditions
Oil Removal Efficiency	80%–95%	Highly efficient when combined with coagulants
Residence Time	10–30 min	Depends on flow rate and bubble size
Typical Application	Offshore discharge, final polishing	Often follows API and hydrocyclones

In IGF systems, gas is mechanically dispersed into the produced water using impellers, eductors, or spargers. The rising gas bubbles adhere to oil droplets and suspended particles, forming a froth layer at the top that is skimmed off. DAF systems, in contrast, use pressurized dissolution of gas followed by rapid pressure release to generate microbubbles (<100 µm), enhancing removal efficiency for smaller particles. Flotation units can remove oil droplets as small as 5–10 µm and are often used as polishing steps before discharge or tertiary treatment [51]. Gas type, bubble size, residence time, and water chemistry (pH, surfactants, temperature) influence their performance.

Primary treatment systems are favored for their simplicity, reliability, and low operational costs. They are designed to handle large volumes of produced water with minimal energy input and mechanical complexity [52, 53]. API separators, hydrocyclones, and flotation units can be easily scaled or arranged in parallel to meet increasing production rates. Moreover, these systems require limited chemical dosing and generate recoverable oil that can be reprocessed, contributing to operational efficiency [53, 54]. Their modular design allows for straightforward integration into existing processing facilities, making them particularly valuable in both onshore and offshore production contexts. Despite their advantages, primary treatment units have inherent limitations due to their reliance on physical separation mechanisms [54, 55, 56]. They are ineffective for removing dissolved salts, metals, and soluble organic compounds such as BTEX, phenols, and PAHs. As a result, the effluent from primary treatment still contains significant amounts of dissolved contaminants, requiring further polishing through secondary and tertiary treatment processes. Additionally, performance may deteriorate under fluctuating flow conditions, high water viscosity, or the presence of stable oil‐water emulsions [57]. Scaling, fouling, and gas entrainment may also reduce equipment efficiency over time if not properly maintained.

Primary treatment remains the cornerstone of produced water management, offering an economical and robust approach for bulk oil and solids removal [57, 58, 59]. The combined application of API separators, hydrocyclones, and flotation units ensures progressive reduction of suspended contaminants, preparing the effluent for more advanced secondary and tertiary treatments [59]. However, these systems are only partially effective, as they do not address dissolved or chemically bound impurities. Hence, their optimization and integration with advanced treatment technologies are essential for achieving regulatory discharge standards and promoting sustainable produced water reuse.

### 3.2 Secondary Treatment of Produced Water (Removal of Residual Oil and Organics)

Primary treatment of produced water (PW) is not a guarantee or final treatment of purity, as there could be finely dispersed oil droplets in the produced water. To completely remove all these impurities, further treatment is carried out, which is the secondary treatment [60]. This removes finely dispersed oil droplets, dissolved hydrocarbons, and organic compounds that remain after primary treatment processes such as gravity separation or hydrocycloning. The impurities in these residues typically range between 10–100 mg/L oil concentration, coupled with some biodegradables and refractory organic matter [61]. To achieve the industrial or environmental standard of effluent discharge of often <10 mg/L oil and grease, secondary or tertiary treatment is necessary [62, 63]. The key technologies commonly employed in this phase include coagulation‐flocculation, dissolved air flotation (DAF), and adsorption‐based filtration media such as activated carbon and walnut shell filters.

#### 3.2.1 Coagulation‐Flocculation

**FIGURE 8:**
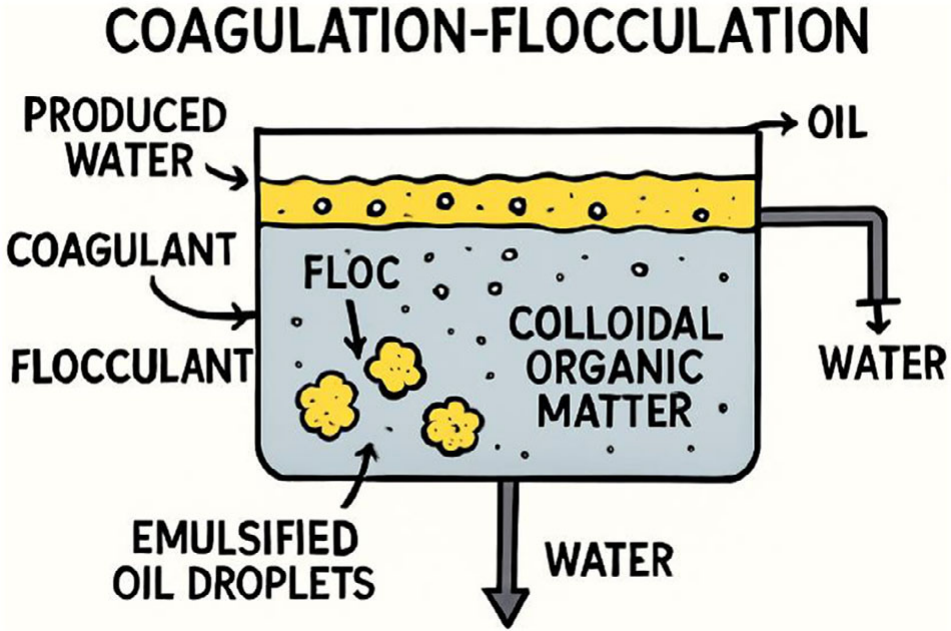
Coagulation‐flocculation units.

The chemical process of destabilizing and aggregating fine colloidal particles and then emulsifying the oil droplets that could not be separated through primary treatment is known as Coagulation‐flocculation [58]. Most often, metallic salt coagulants such as aluminum sulphate (alum, XAl(SO_4_)_2_.12H_2_O), ferric chloride (FeCl_3_), or polyaluminium chloride (PAC) neutralize the surface charges of dispersed oil and organic particles. Once that is achieved, flocculant substances like polyacrylamides (PAMs) or natural biopolymers are added to bridge particles into larger, denser flocs, facilitating separation by sedimentation or flotation. Figure 8 shows a schematic diagram of a coagulation‐flocculation unit [59, 60, 61].

This process is highly effective in removing emulsified oil droplets smaller than 20 µm and colloidal organic matter, including Chemical Oxygen Demand (COD) and Total Suspended Solids (TSS). The typical treatment efficiency ranges between 70 to 95% for oil removal and 60 to 80% for COD reduction, depending on pH control, coagulant dosage, and mixing intensity. Table 7 highlights the performance of coagulation‐flocculation for Produced Water [63, 64]

**TABLE 7:** Typical performance of coagulation‐flocculation for produced water [63, 64].

Parameter	Typical inlet concentration (mg/L)	Effluent after treatment (mg/L)	Removal efficiency (%)	Optimum pH range
Oil & Grease	50–200	5–20	80–95	6.0–8.5
COD	500–1500	200–400	60–80	6.5–8.0
TSS	100–500	20–80	70–90	6.0–8.0

The coagulation–flocculation process offers several advantages that make it attractive for secondary treatment of produced water. It is simple and adaptable for field applications, allowing easy implementation in both onshore and offshore facilities without the need for complex equipment [62]. The process also provides high removal efficiency for fine oil droplets and colloidal particles, ensuring a significant reduction in turbidity and organic load. Moreover, it can be seamlessly integrated before flotation or filtration units, enhancing the overall treatment performance by reducing the load on subsequent processes.

However, the method also has certain limitations. It tends to increase operating costs due to the continuous consumption of chemical reagents such as coagulants and flocculants. Additionally, it produces large quantities of chemical sludge that require proper handling, dewatering, and disposal, which can pose environmental and operational challenges [64, 65, 66]. The process is also sensitive to variations in water chemistry, particularly pH and salinity, which can affect coagulant performance and stability, leading to inconsistent treatment results if not carefully controlled [65].

#### 3.2.2 Dissolved Air Flotation (DAF)

Another widely used technique for the treatment of produced water is the Dissolved Air Flotation (DAF). Dissolved Air Flotation is one of the most widely applied physical separation techniques for secondary treatment of produced water [67, 68]. It utilizes fine air bubbles between 30 to 80 µm, generated by dissolving air under pressure and then releasing it at atmospheric conditions in a flotation tank. The microbubbles attach to hydrophobic oil droplets and suspended solids, forming buoyant aggregates that rise to the surface for skimming.

DAF units are highly effective in removing residual oil‐in‐water emulsions and associated organic matter, especially after coagulation–flocculation pretreatment. The integration of chemical aids significantly improves performance, as coagulated flocs provide suitable surfaces for bubble attachment and enhanced buoyancy. Typical removal efficiencies for oil and grease are 85%–98%, while COD reduction can reach 50%–75% [60, 61, 62].

The dissolved air flotation (DAF) system offers several significant advantages in the secondary treatment of produced water. It is highly effective for removing fine oil droplets and suspended **s**olids, producing a clarified effluent suitable for discharge or further polishing. The technology also features a compact footprint and supports continuous operation, making it ideal for both onshore and offshore applications where space and operational efficiency are critical. In addition, DAF serves as an excellent polishing step following coagulation, enhancing the removal of residual oil and particulate matter that may have escaped earlier separation stages, thereby improving overall treatment performance [61].

However, the DAF process has a few limitations that must be carefully managed. It generally requires high energy input for air pressurization, which can increase operational costs. The system also demands precise control of bubble size and recycle ratio to maintain stable performance and achieve optimal flotation efficiency [65]. Moreover, DAF is less effective in treating dissolved organic compounds, particularly hydrophilic substances, as these do not readily attach to air bubbles, necessitating further treatment through adsorption or advanced oxidation processes to meet stringent effluent standards [66].

#### 3.2.3 Adsorption Media Systems

Adsorption processes are widely used as polishing steps in secondary and tertiary treatment of produced water to remove dissolved organic compounds, trace hydrocarbons, and residual surfactants that persist after flotation. Two common adsorption media are activated carbon and walnut shell filters, both of which utilize high surface area and specific surface chemistry to trap organics either through physical adsorption or surface interactions.

- Activated Carbon Adsorption

Activated carbon (AC), which could be in granular (GAC) form or in powdered (PAC) form, has been an effective removal of impurities, a good adsorbent for removing dissolved organics, phenols, and aromatic hydrocarbons. The mechanism involves Van der Waals forces and hydrophobic interactions that attract organic molecules to the porous carbon matrix. For better results in the treatment of produced water, Granular Activated Carbon (GAC) columns are preferred for continuous operation [66]. This activated carbon adsorption can achieve 70%–95% removal of dissolved organics and up to 99% reduction in oil and grease, depending on carbon type, surface area, and contact time. Regeneration through thermal or chemical methods extends its lifespan, though performance may decline due to fouling by inorganic salts or oil films. Table 8 summarizes Activated Carbon Adsorption Efficiency [67].

**TABLE 8:** Activated carbon adsorption efficiency [67].

Contaminant	Typical inlet (mg/L)	Effluent (mg/L)	Removal (%)	Contact time (min)
Oil & Grease	10–50	0.5–5	90–99	20–40
COD	200–500	50–150	70–85	20–40
Phenols	10–30	1–5	80–95	30–60

The activated carbon adsorption process provides several notable advantages in the secondary treatment of produced water. It offers high efficiency for removing dissolved and refractory organic compounds, resulting in treated effluent suitable for both discharge and reuse. Another key benefit is that the activated carbon media can be regenerated and reused, making it a sustainable option for long‐term operation [69]. However, the process also has certain limitations. It is susceptible to fouling by oils and suspended particulates, which can reduce adsorption efficiency over time. Additionally, regeneration of the carbon media increases operational costs, and the process exhibits limited effectiveness in saline environments, where high ionic strength can interfere with adsorption mechanisms [68, 69].

- Walnut Shell Filters

Walnut shell filters (WSF) are a cost‐effective alternative to activated carbon for polishing produced water, especially in offshore and onshore oilfield facilities. Crushed walnut shells act as a natural medium with moderate surface area and hardness, providing both filtration and mild adsorption. The process involves trapping oil droplets and suspended solids within the shell bed, which can be regenerated by backwashing with air or gas. Walnut shell filters are effective in reducing oil and grease to <5 mg/L and TSS to <10 mg/L, with removal efficiencies typically between 85%–95%. They are particularly advantageous due to their resistance to oil fouling and long media lifespan (often exceeding 5 years) [70]. Table 8 gives the performance of walnut shells as a treatment for produced water (Table 9).

**TABLE 9:** Performance of walnut shell filters in produced water treatment [70].

Parameter	Inlet concentration (mg/L)	Effluent (mg/L)	Removal efficiency (%)	Backwash frequency
Oil & Grease	30–100	3–5	90–95	Every 8–12Hz
TSS	50–150	5–10	85–90	Every 8–12 Hz
COD	200–400	100–200	50–70	Every 8–12 Hz

The walnut shell filter system offers several advantages in produced water treatment, including low maintenance requirements and a long service life, making it a cost‐effective and reliable option for continuous operation. It is highly tolerant to high oil concentrations and can be easily regenerated through simple backwashing without the use of chemicals, ensuring consistent performance and minimal environmental impact [70]. However, the system has some limitations, such as a limited capacity for removing dissolved organics compared to activated carbon, and it requires periodic media cleaning and pressure monitoring to maintain efficiency. Additionally, walnut shell filters are less effective for polar or hydrophilic contaminants, which may necessitate further polishing by adsorption or advanced treatment methods. Table 10 gives a comparison of all the secondary treatments for produced water.

**TABLE 10:** Comparison of secondary treatment technologies for produced water [70].

Process	Primary mechanism	Target contaminants	Typical removal efficiency (%)	Major advantages	Major limitations
Coagulation‐Flocculation	Charge neutralization & aggregation	Emulsified oil, colloids	70–95	Simple, effective, low capital cost	Sludge disposal, chemical cost
Dissolved Air Flotation	Bubble attachment & flotation	Residual oil, solids	85–98	Compact, continuous operation	Energy cost, less effective for dissolved organics
Activated Carbon Adsorption	Surface adsorption	Dissolved organics, phenols	70–99	Excellent polishing, reusable	Fouling, regeneration cost
Walnut Shell Filter	Physical filtration & mild adsorption	Oil droplets, suspended solids	85–95	Long media life, low maintenance	Limited for dissolved compounds

**FIGURE 9:**
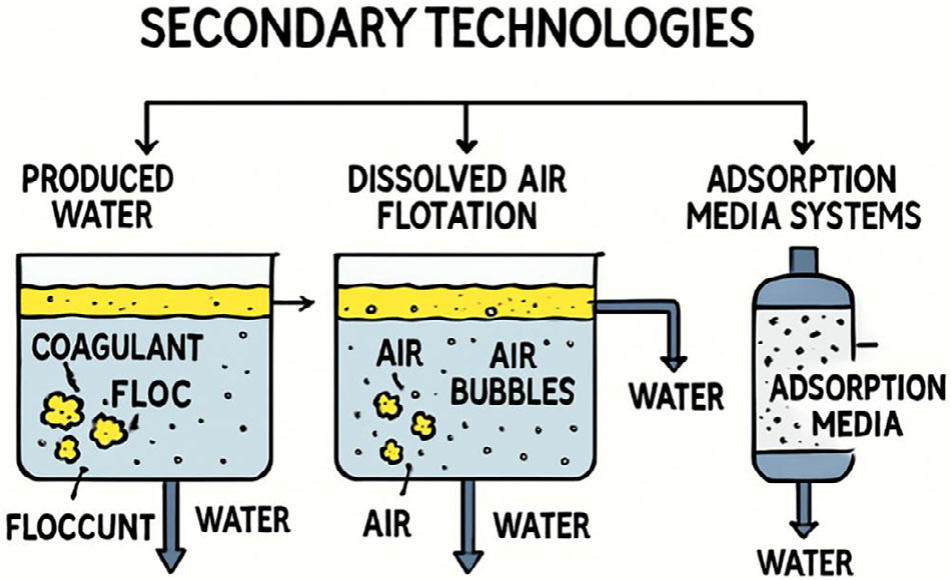
Summarizes secondary techniques.

Secondary treatment plays a crucial role in ensuring that produced water meets discharge or reuse standards by effectively targeting residual oil, emulsions, and dissolved organics that persist after primary separation. Coagulation–flocculation and dissolved air flotation are highly effective for removing fine oil droplets and colloidal matter, while adsorption‐based systems such as activated carbon and walnut shell filters provide polishing functions to achieve near‐zero oil discharge levels [71, 72, 73]. In practice, hybrid systems combining chemical, physical, and adsorption processes offer the best performance, ensuring operational reliability and compliance with environmental regulations. Figure 9 summarizes all the techniques used under the secondary treatment of produced water.

### 3.3 Membrane Processes for Produced Water Treatment

One of the most adaptable and effective technologies in the treatment of produced water in the oil and gas industries is the Membrane. They provide a physical barrier capable of separating suspended solids, emulsified oil, and dissolved ions through size‐exclusion and selective permeability mechanisms [62, 63, 64, 65, 66]. The constituents of produced water are a complex mixture of oil droplets, total dissolved solids (TDS), organic compounds, and inorganic ions, which challenge conventional treatment processes [65]. Membrane processes, including microfiltration (MF), ultrafiltration (UF), Nano‐Filtration (NF), and reverse osmosis (RO), offer a scalable solution capable of producing high‐quality effluent suitable for reuse or discharge. The selection of a particular membrane system depends on the targeted contaminants: MF and UF are primarily used for oil and grease removal, while NF and RO are applied for TDS and dissolved ion reduction [63, 64].

#### 3.3.1 Microfiltration (MF) and Ultrafiltration (UF) for Oil and Grease Removal

The techniques of microfiltration (MF) and ultrafiltration (UF) are pressure‐driven membrane processes that operate at low to moderate pressures, typically 0.1 to 5 bar and are designed to remove suspended solids, oil droplets, and high‐molecular‐weight organic compounds. The pore sizes of MF membranes generally range between 0.1–1 µm, while UF membranes have smaller pores (typically 0.01–0.1 µm) [62]. These characteristics make MF and UF particularly effective in the secondary treatment stage of produced water, providing a polishing step after primary separation and flotation units.

The mechanism of oil and grease removal in MF and UF involves physical sieving and adsorption. Hydrophobic oil droplets are retained on the membrane surface or within the pores, forming a dynamic cake layer that further enhances rejection efficiency but also contributes to fouling [67]. MF/UF systems can achieve oil and grease removal efficiencies exceeding 95–99%, and reduce total suspended solids (TSS) to below 5 mg/L, meeting most discharge requirements. Table 11 compares the performances of MF and UF.

**TABLE 11:** Typical performance of MF and UF systems for produced water [62, 67].

Parameter	MF effluent	UF effluent	Removal efficiency (%)	Operating pressure (bar)
Oil & Grease (mg/L)	3–10	15	95–99	1–3
TSS (mg/L)	5–10	1–5	90–99	1–5
COD (mg/L)	200–400	150–250	40–70	1–5

MF/UF membranes are commonly fabricated from polymeric materials (such as PVDF, PES, or PTFE) or ceramic supports that provide higher chemical and thermal stability. Cross‐flow configurations are often preferred over dead‐end designs to minimize fouling by maintaining shear at the membrane surface. Advantages of MF/UF include their compact design, continuous operation, and high‐quality effluent with low oil content [72, 73]. However, fouling remains a major operational challenge, caused by the deposition of oil droplets, organic macromolecules, and particulates on the membrane surface or within its pores. Frequent backwashing, chemical cleaning, and pre‐treatment (such as coagulation or flotation) are necessary to sustain flux and prolong membrane life.

#### 3.3.2 Nanofiltration (NF) and Reverse Osmosis (RO) for Total Dissolved Solids (TDS) Reduction

While MF and UF effectively remove particulate and emulsified contaminants, nanofiltration (NF) and reverse osmosis (RO) are applied for the removal of dissolved salts, metals, and low‐molecular‐weight organics from produced water [72]. NF membranes typically have pore sizes between 0.5–2 nm, allowing partial removal of monovalent ions and complete rejection of divalent ions such as Ca^2^^+^ and Mg^2^^+^. In contrast, RO membranes have no discernible pores, functioning primarily through a diffusion solution mechanism that can remove up to 99% of TDS. Table 12 presents the performance of nanofiltration and reverse osmosis

**TABLE 12:** Typical performance of NF and RO systems for produced water [72].

Parameter	NF effluent	RO effluent	Removal efficiency (%)	Operating pressure (bar)
TDS (mg/L)	500–1500	50–200	80–99	10–70
COD (mg/L)	100–300	50–100	60–90	10–70
Sulphate (mg/L)	<50	<5	95–99	10–70
Oil & Grease (mg/L)	<2	<1	99	10–70

NF and RO systems are typically deployed as tertiary treatment units, often after MF/UF pre‐filtration to prevent membrane fouling. The treated effluent from RO systems can meet stringent environmental standards or be reused for injection and irrigation purposes, depending on salinity levels and regulatory requirements. One major advantage of NF/RO is their ability to produce high‐purity water with significantly reduced salinity and organic content [66]. However, despite all these advantages, membrane scaling, fouling, and high salinity limits pose major challenges. Scaling occurs due to the precipitation of sparingly soluble salts (e.g., CaCO_3_ and CaSO_4_), while fouling is caused by organic matter and colloidal residues. Moreover, high salinity levels >50 000 mg/L TDS increase osmotic pressure, thus requiring higher energy input and specialized materials to prevent degradation [66, 67, 68].

#### 3.3.3 Operational Issues: Fouling, Scaling, and High Salinity Limits

**FIGURE 10:**
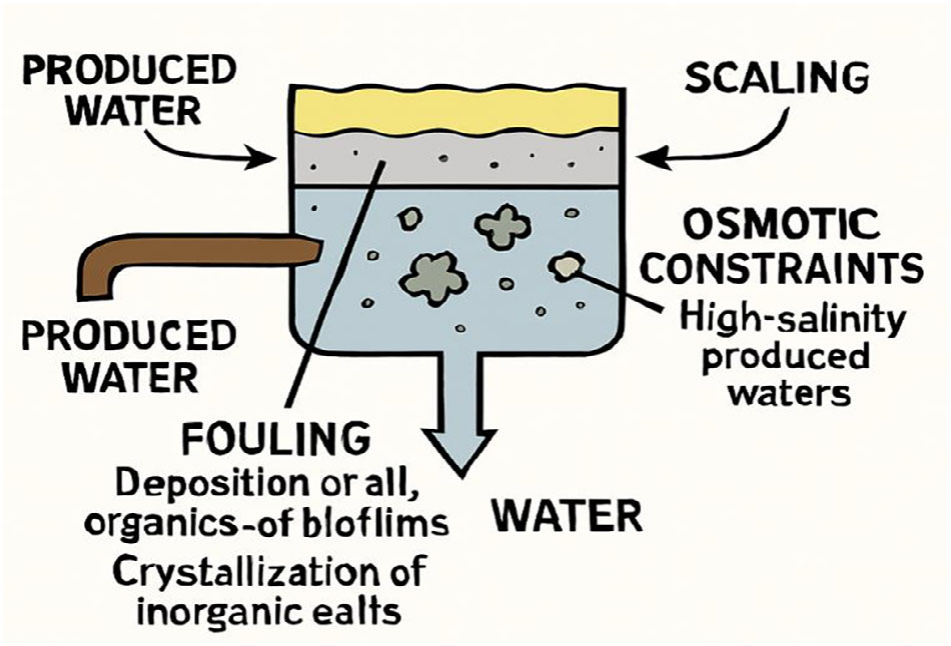
Limitations of the membrane system.

The main operational limitations of membrane systems in produced water treatment are fouling, scaling, and osmotic constraints. Fouling occurs due to the deposition of oil, organics, or biofilms on the membrane surface, leading to flux decline and increased transmembrane pressure [72]. Scaling results from the crystallization of inorganic salts such as calcium carbonate or barium sulfate within the membrane pores, which reduces permeability and may cause irreversible damage (Figure 10).

In high‐salinity produced waters, the osmotic pressure differential across NF or RO membranes can exceed the practical operating range, leading to reduced water flux and increased energy demand. Therefore, pretreatment such as UF or chemical softening with periodic cleaning protocols is essential for sustainable operation.

### 3.4 Technological Advances

Recently, produced water treatment has developed significantly and improved in performance and reliability, especially the membrane systems in produced water treatment, addressing traditional challenges related to fouling and high salinity. There are a few notable advances, such as the ceramic membranes, anti‐fouling surface coatings, and hybrid membrane‐biological systems [71, 72, 73].

#### 3.4.1 Ceramic Membranes

Ceramic membranes made from materials such as alumina, zirconia, or titanium oxide have gained attention due to their high mechanical strength, thermal stability, and resistance to chemical and oil fouling. Unlike polymeric membranes, ceramic variants tolerate extreme pH and temperature conditions (up to 300°C), making them ideal for offshore and refinery applications. They offer longer lifespans and can be cleaned aggressively with acids or alkalis. Though capital costs are higher, their durability and stable flux performance often lead to lower lifecycle costs [72].

#### 3.4.2 Anti‐Fouling Coatings and Surface Modifications

Another significant advancement involves anti‐fouling surface coatings such as hydrophilic polymer grafts, zwitterionic materials, and nanocomposite layers, for instance, TiO_2_, SiO_2_. These coatings enhance water wettability, reducing the adhesion of oil and organic matter to the membrane surface. Studies have demonstrated that anti‐fouling modified membranes can reduce flux decline by 30%–50% compared to unmodified polymeric membranes. This extends cleaning intervals and improves process economics [71].

#### 3.4.3 Hybrid Membrane‐Biological Systems

Hybrid membrane‐biological systems combine the physical separation ability of membranes with the biodegradation capacity of microorganisms. For instance, membrane bioreactors (MBRs), integrate UF membranes with aerobic or anaerobic biological treatment. This configuration allows simultaneous removal of organic pollutants and suspended solids, achieving COD reductions of 90%–95% and TDS reductions of up to 70% when coupled with NF/RO units. Hybrid systems also minimize sludge generation and enhance water recovery for reuse [72]. Table 13 gives a comparison of membrane processes.

**TABLE 13:** Comparison of membrane processes for produced water treatment [72].

Process	Pore size	Target contaminants	Efficiency (%)	Pressure (bar)	Key limitations
MF	0.1–1 µm	Suspended solids, large oil droplets	90–95	1–3	Fouling, limited by dissolved solids
UF	0.01–0.1 µm	Emulsified oil, macromolecules	95–99	1–5	Organic fouling requires cleaning
NF	0.5–2 nm	Divalent ions, small organics	80–95	10–30	Scaling, moderate energy demand
RO	<0.5 nm	Monovalent ions, TDS	95–99	20–70	High pressure, sensitive to fouling

Membrane processes represent a vital component of modern produced water treatment, providing superior separation efficiency, modularity, and compactness compared to conventional methods. MF and UF membranes effectively remove oil, grease, and suspended solids, while NF and RO systems enable significant reductions in dissolved salts and organic matter, producing water suitable for reinjection or reuse [74, 75]. However, the practical challenges of fouling, scaling, and high energy demand under high‐salinity conditions remain major operational barriers. Emerging technologies such as ceramic membranes, anti‐fouling coatings, and hybrid membrane‐biological systems are addressing these challenges by enhancing membrane resilience, reducing maintenance frequency, and improving overall performance. As research advances, the integration of these innovative systems is expected to play a central role in achieving sustainable and cost‐effective produced water management in the oil and gas sector [74, 75, 76, 77].

### 3.5 Thermal Processes Treatment for Produced Water

Produced water from oil and gas exploration contains a substantial amount of dissolved solids, organics such as hydrocarbons and surfactants, suspended solids, scaling ions (Ca^2^^+^, Mg^2^^+^, SO_4_^2^^−^), and sometimes elevated temperature and oil content [72]. When the feed of total dissolved solids or complex chemistry prevents economic treatment by pressure‐driven membranes (RO) or when zero liquid discharge (ZLD) or near‐ZLD recovery is required, thermal or phase‐change processes are the preferred route because they separate water as vapor and leave salts and non‐volatile organics in the concentrate/residue [73]. Modern literature and field reviews emphasize that thermal methods remain the “fallback” solution for brines with very high salinity or oil/organic loading that foul membranes rapidly [72].

#### 3.5.1 Distillation and Evaporation—Principles and Variants

Distillation and evaporation processes for produced water all rely on phase change: heat is supplied to the liquid to vaporize water; the vapor is then condensed to yield product water. In simplest terms, the unit operations include (a) a heater/evaporator where water is boiled or flashed, (b) a vapor‐liquid separator to collect vapor, and (c) a condenser producing distillate. Variants include single‐effect flashing/evaporation (one stage), multi‐effect evaporation (MEE, cascaded effects operating at successively lower pressures), and thermal‐vacuum distillation, where reduced pressure lowers boiling temperature [66, 67, 72]. Flash distillation is used when there is sufficient sensible heat, while contact‐type evaporators (forced circulation, falling film, or rising film) are used where fouling/scaling risk must be managed.

Distillation and evaporation processes offer several advantages in the treatment of produced water. They enable virtually complete removal of non‐volatile salts and most organics that do not co‐distil, ensuring a high level of purification. These processes are also highly robust to the presence of oil and suspended solids, as these contaminants remain with the brine, reducing the extent of pre‐treatment required compared to membrane‐based technologies such as reverse osmosis (RO). Their reliability has been proven in zero liquid discharge (ZLD) systems and in various industries that handle high‐TDS (total dissolved solids) streams. However, the main limitation of distillation and evaporation lies in their high energy demand, since significant heat input is required to overcome the large latent heat of vaporization [69, 70, 71, 72, 75, 78]. Additionally, scaling and fouling of heat‐transfer surfaces caused by calcium carbonate, sulfates, silica, and polymeric organics can significantly impair efficiency, necessitating rigorous scaling control through anti‐scalants, blowdown management, or crystallizer integration. In extremely saline brines, increased viscosity and salt crystallization further complicate pumping and heat‐transfer operations, often requiring specialized solids‐handling equipment such as crystallizers and dryers to maintain continuous operation [75].

#### 3.5.2 Mechanical Vapor Recompression (MVR)

Mechanica Vapor recompression (MVR) is a thermally driven process that captures vapor produced by evaporation and re‐compresses it mechanically by a centrifugal or positive‐displacement vapor compressor [74]. Vapor pressure and temperature are increased by compression, so that it can act as the heating medium for further evaporation for the reuse of the latent heat. In practice, an evaporator produces vapor which is drawn into the compressor; the compressed vapor then condenses on the evaporator's heat transfer surface, supplying heat for additional evaporation (single‐ or multi‐effect arrangements are possible). This recycles the latent heat and greatly reduces external heat input (fuel or steam) compared with direct‐fired or steam‐driven evaporators [75].

##### 3.5.2.1 Strengths of MVR for Produced Water Treatment

- Much higher energy efficiency (lower external thermal energy) than single‐effect evaporation because the compressor supplies mechanical work (electrical) instead of steam generation.
- Electrical energy (for the compressor) can be cheaper and be supplied from local generation; efficiencies scale favorably at moderate capacities.
- Good candidate where low‐grade heat is unavailable but electricity is present.

##### 3.5.2.2 Design and Operational Issues

**FIGURE 11:**
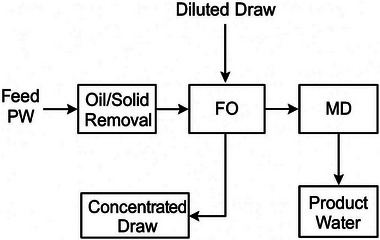
Simple MVR process sketch (textual diagram).

- Compressor reliability and maintenance: vapors may carry organics, salts or droplets that can damage compressors; effective vapor–liquid separation and droplet entrainment control are needed.
- For extremely high concentrations and crystallizing salts, heat transfer surfaces will foul, so MVR systems require design features (e.g., falling‐film evaporators, chemically cleaned surfaces) to manage scaling.
- There is a tradeoff: MVR shifts the energy burden from thermal to electrical; depending on the electricity carbon intensity and cost, life‐cycle impacts must be assessed. Recent exergy and energy analyses in the literature quantify these tradeoffs and recommend hybrid schemes or multi‐effect + MVR combinations to optimize performance (Figure 11).

##### 3.5.2.3 Suitability for High‐TDS Brines and Energy Intensity

Thermal processes excel at very high TDS because vapor‐liquid separation is independent of osmotic pressure and membrane permeability limits. Typical pressure‐driven membranes (RO) are limited to ∼70–80 g/L (70 000–80 000 mg/L TDS) in practical operation; beyond this, osmotic pressure and scaling make RO uneconomic or impossible [62]. Thermal systems, by contrast, have been used for brines in the range of hundreds of g/L (approaching saturation) when robust crystallization and solids handling are included. This makes them the technology of choice for ZLD or near‐ZLD treatment of produced water where resource recovery or strict discharge limits apply.

However, thermal processing is energy‐intensive because the latent heat of vaporization of water (∼2257 kJ/kg at 100°C) must be supplied or recovered. Even with MVR or multi‐effect designs, the *specific energy consumption* (SEC) is typically much higher than RO for the freshwater per cubic meter produced, though energy per mass of salt removed may be competitive at very high concentrations [61, 62]. Energy reductions come from:

- Using MVR to recycle latent heat (electrical work to the compressor),
- Multi‐effect or multi‐stage configurations to reuse heat Across stages, and
- Hybridization (pre‐concentration by RO/membranes, finishing by thermal) so that thermal units treat smaller, concentrated flows. Life‐cycle and techno‐economic studies in the last five years show that hybrid membrane + thermal trains are often the lowest‐cost path to ZLD.

### 3.6 Recent Innovations

Although FO and MD are not strictly “thermal” in the classical sense, both are osmotic**/**thermally‐driven membrane processes that bridge the gap between membranes and thermal phase change, and they are attracting intense research as lower‐energy or fouling‐tolerant alternatives for produced water.

#### 3.6.1 Forward Osmosis (FO)

Forward Osmosis uses an osmotic pressure gradient (a concentrated draw solution) to pull water across a semi‐permeable membrane from the feed into the draw [71]. The diluted draw is subsequently regenerated to recover product water and recover the draw solute. FO's advantages for produced water include high fouling tolerance (lower hydraulic shear across the membrane) and the ability to handle high TDS feeds as the osmotic driving force is provided by the draw rather than feed pressure. Current research focuses on: (a) novel draw solutes that are easy to recover (volatile or thermolabile compounds like ammonium bicarbonate, thermos‐responsive ionic liquids), (b) robust FO membranes with low internal concentration polarization (ICP), and (c) hybrid FO‐MD or FO‐RO trains where FO pre‐concentrates the feed and reduces load on downstream thermal or pressure units [75, 76, 77]. Recent reviews report promising lab‐scale fluxes and pilot trials, but draw recovery and overall system energy for draw regeneration remain the critical bottleneck.

#### 3.6.2 Membrane Distillation (MD)

Membrane Distillation MD is a thermally driven membrane process that uses a hydrophobic microporous membrane. A vapor pressure gradient (created by temperature difference) drives water vapor from the hot feed side across the membrane pores to the cold permeate side, where it condenses. MD benefits from operation at low to moderate temperatures (30–90°C), tolerates very high salinities, and is less sensitive to fouling than RO because salts remain on the feed side. MD pairs well with low‐grade heat sources (waste heat, solar thermal) and can be integrated with MVR or with FO in hybrid systems [79, 80]. Recent technical advances include novel membrane materials with better wetting resistance, anti‐fouling surface modifications, improved module designs (air‐gap MD, vacuum MD, direct contact MD), and combinations with crystallization units for ZLD. The MD literature indicates substantial improvements in membrane lifetime, but scale‐up and cost competitiveness remain active research areas [79].

#### 3.6.3 FO/MD and Hybrid Concepts

Hybrid schemes aim to exploit the low‐fouling pre‐concentration of FO with the high‐recovery, thermally tolerant MD. For example, FO first removes a large fraction of water into a draw; the diluted draw is then desalinated by MD (or other thermal/regeneration means) to produce product water and a reconcentrated draw. Alternatively, RO can be used where feasible as a pre‐concentrator with MD/MVR finishing the residual brine to ZLD. Hybridization is a strong trend in recent reviews and pilot reports because it reduces the volume of the thermal unit and thus the total thermal energy required (Table 14).

**TABLE 14:** Comparative Summary [75, 76, 77, 79, 80].

Technology	Driving force	Typical feed TDS suitability	Energy form (dominant)	Fouling and organics tolerance	Typical application/remarks
Single‐effect evaporation/distillation	Heat (phase‐change)	High (up to saturation)	Thermal (steam/fuel)	Moderate‐high (heat surfaces foul)	Simple, robust, high‐energy
Multi‐effect evaporation (MEE)	Cascaded low‐grade heat reuse	High	Thermal, reduced per m^3^ vs single‐effect	Same scale/fouling concerns	Good for larger plants with steam
MVR (single/multi)	Mechanical recompression of vapor	Very high	Electrical (compressor) + small thermal	Sensitive to carryover; good if adequate separation	Energy‐efficient thermal option
Membrane Distillation (MD)	Vapor pressure (temperature diff.)	Very high	Low‐grade thermal	Better than RO, membrane wetting risk	Good with waste heat/solar
Forward Osmosis (FO)	Osmotic gradient (draw)	Moderate‐high (depends on membrane)	Depends (draw regeneration dominates)	High fouling tolerance	Good as a pre‐concentrator; draw recovery key
Hybrid (RO + thermal / FO + MD / MVR + MD)	Mixed	Very high	Optimized mix	Optimized by the separation of duties	Often, the lowest cost for ZLD scenarios

Thermal processes (evaporation, distillation and MVR) remain indispensable tools for treating produced water when RO or conventional membranes are infeasible, particularly for very high‐TDS brines and ZLD objectives. MVR reduces thermal energy demand by recycling latent heat electrically and is an attractive route where electricity is available; MD and FO are newer routes that can reduce fouling and exploit low‐grade heat or provide an effective pre‐concentration stage. The research agenda should prioritize: (a) pilot‐scale hybrid systems (FO/RO + MD/MVR) under representative produced‐water chemistries, (b) membrane materials and anti‐fouling strategies for MD/FO, (c) reliable draw‐recovery routes for FO, and (d) life‐cycle energy/carbon assessments of alternatives under site‐specific conditions. Recent review literature and pilot studies indicate vigorous progress, but scale‐up and integrated techno‐economic demonstration remain the gating challenges for wider adoption [79, 80, 81].

### 3.7 Biological Treatment of Produced Water

Another treatment of produced water is the biological method. The Biological treatment is one of the most attractive options for removing biodegradable organic matter from produced water when oil and salts are at manageable levels. Bioreactors ranging from conventional activated sludge systems to attached‐growth biofilm reactors and membrane bioreactors (MBRs) use microbial metabolic activity to oxidize hydrocarbons, soluble biochemical oxygen demand (BOD/COD), and some dissolved organics into simpler compounds (CO_2_, biomass, inorganic ions). MBRs are particularly promising for produced water because they combine biological degradation with membrane separation, enabling higher biomass concentrations, longer solids retention times (SRT), and good solids‐liquid separation even when the feed contains fine emulsified oil [37, 38]. However, salt stress, oil toxicity, and inhibitory organics commonly present in produced water impose constraints on biological performance; acclimation with halotolerant or halophilic consortia and careful control of operating conditions (SRT, temperature, nutrient balance) are often required to achieve stable COD removal. Membrane‐based bioreactors have been reviewed extensively and show good removal when TDS is moderate, yet fouling and salinity effects remain significant operational challenges [46].

#### 3.7.1 Bioreactor Configurations and Mechanisms

Several bioreactor configurations have been applied in the treatment of produced water, each with distinct operational advantages and limitations. Suspended‐growth systems, such as the conventional activated sludge process, offer high mass transfer rates and are well‐established in wastewater treatment [82]. However, their use for produced water is often constrained by washout of microbial populations and filamentous growth caused by oil contamination.to ensure proper and reliable stable performance, the removal of oil and solids before the use of biological treatment through flotation or physical separation stages is necessary [79, 80].

**FIGURE 12:**
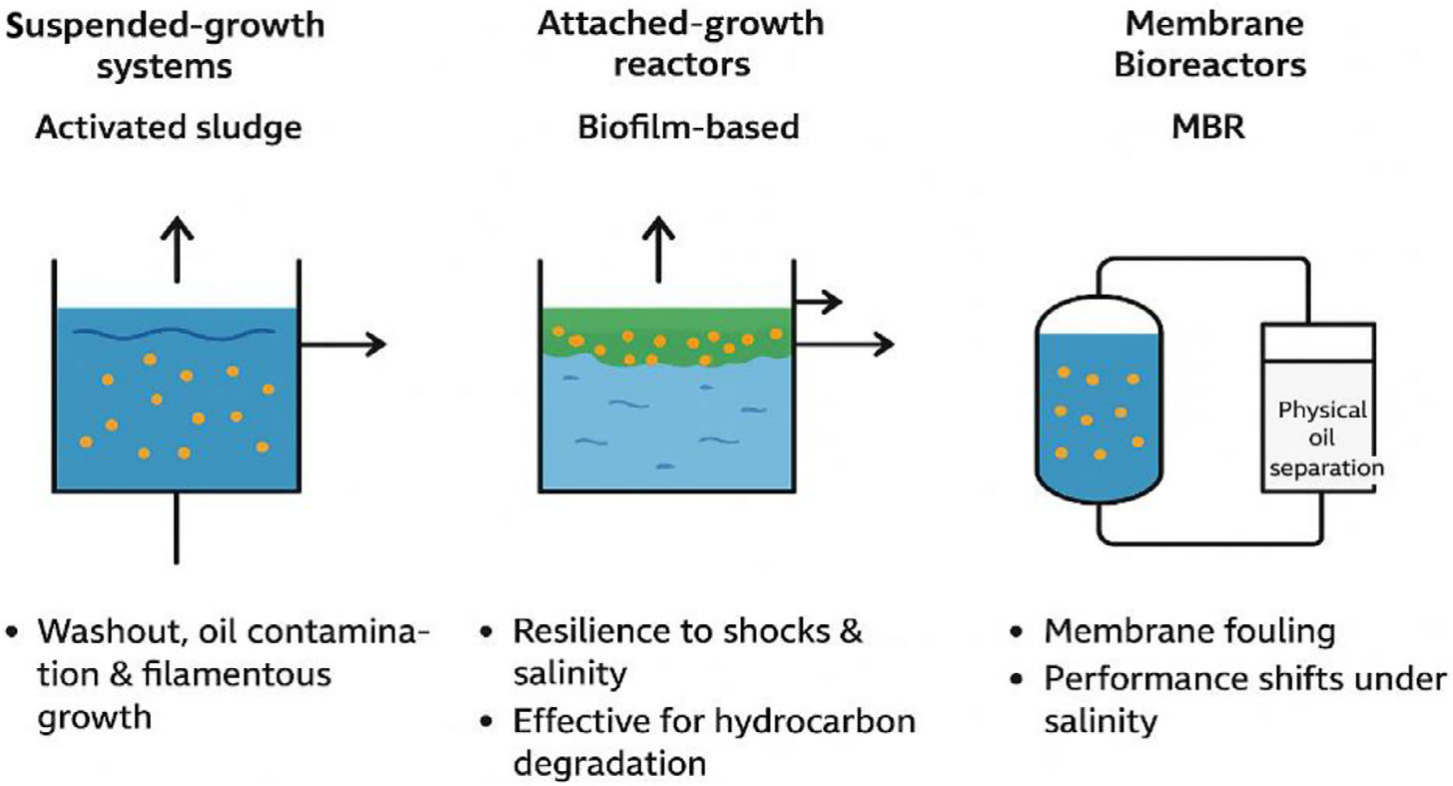
Bioreactor with a membrane.

Before the fluctuation, the attachment of the saline condition, biofilm‐based reactors, including trickling filters and moving bed biofilm reactors (MBBR), provide an alternative that enhances resilience under fluctuating and saline conditions. In these systems, microorganisms attach to media surfaces, forming biofilms that create protective microenvironments, reducing exposure to toxic shocks and high salinity in the bulk liquid. This configuration supports hydrocarbon degradation of microbial communities and demonstrates better tolerance to variations in hydraulic and organic loading, making it particularly suitable for treating complex produced water streams [81, 82, 83]. Reactors like membrane bioreactors (MBRs) represent a more advanced configuration, with the integration of biological degradation with membrane‐based solid‐liquid separation (using microfiltration or ultrafiltration). This integration allows decoupling of hydraulic and solids retention times, which enhances organic degradation and ensures consistent effluent quality within a compact footprint [82]. Despite these advantages, challenges such as membrane fouling, often intensified by oil and suspended solids and performance shifts under saline conditions persist. Recent studies emphasize the development of hybrid MBR systems incorporating pre‐treatment steps, such as physical oil separation, to safeguard the membrane and improve long‐term stability and treatment efficiency. Figure 12 illustrates the process of a bioreactor with a membrane configuration.

#### 3.7.2 Microbial Ecology, Salinity, and Adaptation

Produced water typically presents elevated salinity and sometimes toxic hydrocarbons, which select for halotolerant/halophilic microbes. Several studies show that biological COD removal declines as salinity increases, with notable performance reduction above ∼30–50 g/L TDS in many conventional systems, though targeted enrichment of halophiles (e.g., Halomonas spp. and other salt‐adapted strains) or gradual acclimation can improve resilience. Strategies to improve salt tolerance include bioaugmentation with halophiles, use of biofilm/attached‐growth systems that buffer microbes from bulk salinity, and operating at longer SRT to favor slower‐growing, salt‐tolerant degraders [81, 82, 83, 84]. These biological measures are critical if a biologically based unit will serve as more than a polishing step.

### 3.8 Advanced Oxidation Processes (AOPs)

AOPs generate highly reactive species (chiefly hydroxyl radicals, •OH) that non‐selectively oxidize and mineralize recalcitrant organic contaminants, including many petroleum‐derived compounds, phenolics, and other hard‐to‐degrade molecules. For produced water, especially where residual dissolved organics or trace contaminants remain after bulk removal, AOPs provide powerful polishing or pre‐treatment for downstream biological or membrane systems. The main AOP families relevant to produced water include ozonation, photocatalysis, and Fenton‐type processes.

#### 3.8.1 Ozonation

Ozonation (O_3_) oxidizes organics directly and indirectly (via •OH produced during O_3_ decomposition). It is effective for breaking down complex aromatics and for improving biodegradability (increasing BOD/COD ratio) so that subsequent biological polishing is more effective [66, 67]. Implementation for produced water often uses ozone contactors with off‐gas destruction, and careful dose control is required because ozone reacts with radical scavengers (e.g., chloride, bicarbonate) and can generate bromate or other by‐products when bromide is present. Ozonation is attractive for its rapid kinetics and ability to treat recalcitrant molecules, but in high‐salinity matrices, radical scavenging and secondary by‐product formation reduce efficiency and require careful monitoring [69, 78].

#### 3.8.2 Photocatalysis (UV/TiO_2_ and Related Systems)

Photocatalysis employs a semiconductor (commonly TiO_2_) activated by UV light to produce electron–hole pairs that generate reactive radicals (OH, superoxide). Photocatalytic AOPs can achieve deep mineralization and are particularly useful for low‐volume streams or for targeted removal of priority contaminants. Photocatalysis scales from bench to pilot but faces challenges in produced water: UV attenuation by suspended solids and colored organics, catalyst fouling, and reduced radical lifetime in high ionic‐strength waters [78]. Advances in catalyst materials (doped TiO_2_, immobilized catalyst supports) and reactor engineering (contactors, flow‐through designs) are active research areas to improve efficacy under saline conditions [60].

#### 3.8.3 Fenton and Fenton‐Like Processes

The classical Fenton reaction (Fe^2^^+^ + H_2_O_2_ → Fe^3^^+^ + OH + OH^−^) efficiently generates hydroxyl radicals under acidic conditions and rapidly degrades many organics. Fenton‐like adaptations (heterogeneous catalysts, photo‐Fenton, chelated iron) broaden applicability and can reduce sludge and iron handling issues [70, 71]. For produced water, Fenton processes can provide rapid oxidation but are sensitive to matrix components that scavenge radicals (high chloride, bicarbonate) and to pH control issues. Moreover, iron residuals and reaction by‐products require post‐treatment [72]. Recent reviews highlight Fenton's utility in polishing applications where volume is limited and where subsequent separation (adsorption or biological polishing) can handle by‐products.

However, there are some limitations to this kind of treatment. High salinity in produced water has several negative impacts on both biological systems and AOPs. For biological units, salinity exerts osmotic stress on microbes, suppressing activity unless halophilic organisms are selected or acclimated; performance typically drops as TDS rises above tens of grams per liter. For AOPs, particularly those relying on hydroxyl radicals, dissolved ions such as chloride, bicarbonate, and carbonate act as radical scavengers, dramatically reducing oxidative efficiency and increasing required reagent doses or energy inputs [40, 41, 42]. Additionally, bromide in produced water can form regulated disinfection by‐products (bromate during ozonation). Therefore, biological treatment and AOPs are most effective as polishing steps applied after oil removal and bulk solids separation, and often after partial desalination or volume reduction (e.g., via RO when feasible) to minimize salinity‐related impacts. Hybrid treatment trains, biological + AOP polishing, frequently represent the best balance between cost, energy, and effluent quality. Table 15 shows the comparison between the biological and advanced Oxidation Process (AOP) [7, 49, 50, 69, 70, 78].

**TABLE 15:** Comparison of biological and AOP methods for produced water [7, 49, 50, 69, 70, 78].

Method	Target contaminants	Typical TDS tolerance	Main strengths	Main limitations
Suspended‐growth bioreactor (activated sludge)	Readily biodegradable organics, oils (after separation)	Low–moderate (<30–50 g/L)	High biodegradation rates, established tech	Sensitive to salinity and oil shocks
Attached‐growth / MBBR	Hydrocarbons, oils, BOD	Moderate (improved resilience)	Robust to shocks, compact	Biofilm control, sometimes slower
Membrane Bioreactor (MBR)	Suspended solids + biodegradable organics	Moderate (membranes sensitive to fouling)	High effluent quality, small footprint	Membrane fouling, salinity effects
Constructed Wetland	BOD, TSS, hydrocarbons (polishing)	Low–moderate; halophyte designs extend range	Low energy, passive, co‐benefits	Land area, seasonal variability, and plant stress at high salinity
Ozonation (AOP)	Recalcitrant aromatics, color, toxics	Reduced efficacy at high salt; radical scavenging	Fast oxidation improves biodegradability	Bromate formation, high reagent cost in saline matrices
Photocatalysis (UV/TiO_2_)	Recalcitrant organics, micropollutants	Challenged by high ionic strength and turbidity	Deep mineralization potential	UV attenuation, catalyst fouling, energy costs
Fenton / Photo‐Fenton	Rapid oxidation of organics	Affected by radical scavengers; pH sensitive	Fast reaction rates; inexpensive reagents	Iron sludge, pH control, reduced effect in high‐salt waters

### 3.9 Case Study

#### 3.9.1 Onshore Produced Water Reuse via Hybrid Membrane System (Saudi Arabia)

In Saudi Arabia, an onshore gas production facility implemented a hydrocyclone UF‐RO evaporator system to effectively treat and manage produced water with an estimation of about 25 000 mg/L of total dissolved solids (TDS). The installed hydrocyclone managed the resulting oil production in the produced water by reducing oil from 200 mg/L to <30 mg/L, followed by UF, which brought turbidity below 1 NTU. Also, RO desalination achieved 75% water recovery, producing permeate with approximately 500 mg/L TDS suitable for steam generation and irrigation. The remaining concentrate was fed to a mechanical vapor recompression (MVR) evaporator to achieve ZLD. Optimization through energy recovery (heat integration between RO reject and evaporator feed) reduced total energy demand by 15%, while online fouling monitoring decreased chemical cleaning frequency by 40%.

#### 3.9.2 Offshore Platform Compact Integration (North Sea)

Flotation unit (CFU) ceramic UF system for produced water polishing before discharge. The system Due to space and weight constraints, a North Sea offshore platform deployed a compact hydrocyclone, compactly reducing dispersed oil from 150 mg/L to below 10 mg/L, meeting OSPAR discharge limits. The modular UF unit operated at 60°C with minimal fouling due to integrated air backwash and hydrophilic ceramic membranes. Computational fluid dynamics (CFD) optimization of flow distribution across the UF modules improved flux uniformity by 12%, reducing backwash frequency and footprint.

#### 3.9.3 Thermal‐Membrane Hybrid for ZLD (China)

A high‐TDS (>100 000 mg/L) produced water stream from a coal‐bed methane facility was treated using a UF‐RO‐MVR evaporator‐crystallizer. The RO achieved 35% recovery, reducing feed load to the evaporator. Total system recovery of about 92% can be achieved by incorporating condensate from the evaporator back into the UF feed. 22% of the thermal energy from the heat compressor discharge can be saved by preheating the MVR. The hybrid configuration demonstrated robust operation even under feed total dissolved solids (TDS) fluctuations, emphasizing that energy recovery and feedback control are central to optimization.

To optimize and manage produced water, there is need for proper and intelligent integration of specific technologies across scales of operating condition and not the selection requires more than the selection of individual techniques. Hybrid trains such as hydrocyclone‐UF‐RO‐thermal evaporator exemplify the synergy between mechanical separation, membrane filtration, and thermal concentration. A robust pretreatment technique holds the key to a sustainable operation of scaling‐fouling control and process integration that reuses energy and water within the system. AI‐driven design and monitoring innovation, modular compact design**s**, and low‐grade heat utilization for energy efficiency. As global regulations stiffen and water reuse becomes integral, optimized hybrid systems represent the frontier for reliable, cost‐effective, and environmentally sound produced water management.

## 4 Conclusions

The objectives of this review are to critically synthesize current produced water treatment technologies from a systems‐level perspective rather than as isolated unit operations; to evaluate optimization drivers and constraints, including pretreatment requirements, energy integration, fouling and scaling control, and operational flexibility; to assess the suitability of standalone and hybrid treatment trains for disposal, reuse, and zero‐liquid‐discharge applications; and to identify key research and implementation gaps related to sustainability metrics, life‐cycle impacts, and digital optimization This review demonstrates that effective produced water management cannot be achieved through standalone treatment technologies, due to the extreme variability in water chemistry, flow rates, and regulatory targets across oil and gas operations. Across the literature, hybrid treatment trains integrating primary oil‐water separation, targeted pretreatment, membrane‐based desalination, and thermal or oxidative polishing consistently provide superior robustness, water recovery, and operational resilience. The synthesis further shows that system performance is governed less by individual unit efficiency and more by pretreatment adequacy, fouling and scaling control, energy integration, and adaptability to fluctuating feed compositions. Consequently, optimization in produced water treatment is inherently a system‐level challenge rather than a component‐level problem

The design and optimization of produced water treatment systems demand an integrated approach that combines scientific innovation, process engineering, and environmental management. From conventional separators and flotation units to sophisticated hybrid trains incorporating biological, oxidative, and membrane‐based systems, the evolution of produced water technology reflects an ongoing commitment to efficiency, compliance, and sustainability. Efficient management requires balancing operational feasibility with environmental safety, ensuring that each treatment stage contributes to the overall system performance. The integration of different interdisciplinary research, such as materials science, process control, microbiology, and energy systems, will be key to overcoming present challenges. Optimization of produced water management not only mitigates ecological impact but also transforms a major waste stream into a valuable resource, reinforcing the oil and gas industry's transition toward cleaner and more sustainable operations.

For industry, the findings highlight the need to shift from technology‐centric selection toward integrated design frameworks that align treatment objectives with reuse pathways, energy availability, and site‐specific constraints. Pretreatment design and operational flexibility emerge as critical determinants of long‐term reliability and cost control, particularly for membrane‐ and thermal‐based systems. From a regulatory perspective, the results support performance‐based discharge and reuse standards that incentivize system optimization, water recovery, and environmental impact reduction rather than prescriptive technology mandates. Clear regulatory signals regarding reuse quality, brine disposal, and lifecycle emissions are essential to accelerate the adoption of optimized hybrid treatment systems and circular water management practices.

Despite significant technological advances, several critical gaps remain. First, there is a lack of full‐scale and long‐term performance data for emerging hybrid systems, particularly under highly saline and compositionally variable conditions. Second, optimization frameworks integrating techno‐economic analysis, life‐cycle assessment, and carbon footprint metrics are still scarce, limiting informed decision‐making at the system level. Third, while digital tools such as real‐time monitoring, predictive control, and digital twins show promise, their application in produced water treatment remains largely conceptual and unvalidated. Future research should prioritize pilot‐scale demonstrations, data‐driven optimization frameworks, and integrated sustainability assessments to support scalable, resilient, and regulation‐compliant produced water reuse and resource recovery strategies.

## Conflicts of Interest

The authors declare no conflicts of interest.

## Data Availability

The authors have nothing to report.
